# Potentially inappropriate medications in older outpatients with depression: a cross-sectional study comparing the Beers criteria and the Chinese criteria

**DOI:** 10.3389/fpsyt.2026.1915277

**Published:** 2026-09-03

**Authors:** Hong Jiang, Xiaojuan Zhou, Pinpin Feng, Zhongni Xia, Wenjuan Yang, Bing Han, Meiling Zhang, Qinqin Zhao

**Affiliations:** 1 Department of Pharmacy, Tongde Hospital of Zhejiang Province, Hangzhou, China; 2 Department of Pharmacy, Zhejiang Academy of Traditional Chinese Medicine, Hangzhou, China

**Keywords:** potentially inappropriate medications, Beers criteria, Chinese criteria, depression, older

## Abstract

**Objective:**

To determine the prevalence of PIMs and identify associated factors in older Chinese outpatients with depression, using a combined evaluation based on the 2023 Beers criteria and the 2017 Chinese criteria.

**Methods:**

A cross-sectional study was conducted at a provincial tertiary psychiatric hospital in China, enrolling older outpatients (≥ 65 years) diagnosed with depression. PIM status was identified according to the 2023 Beers criteria and the 2017 Chinese criteria. Logistic regression analyses were performed to identify factors associated with PIMs. Interaction, subgroup, and sensitivity analyses were also performed.

**Results:**

Among 3,155 eligible patients (mean age 74.22 ± 6.70 years), PIM prevalence was 92.71% under the Beers criteria and 65.13% under the Chinese criteria. Under both criteria, quetiapine, benzodiazepines and sedative-hypnotics were the most frequently identified as PIMs. Female sex (Beers: aOR = 1.57, 95% CI: 1.16-2.12; Chinese: aOR = 1.44, 95% CI: 1.20-1.73), polypharmacy (Beers: aOR = 2.43, 95% CI: 2.08-2.85; Chinese: aOR = 2.51, 95% CI: 2.30-2.74), and comorbid sleep disorders (Beers: aOR = 1.79, 95% CI: 1.27-2.51; Chinese: aOR = 3.67, 95% CI: 2.99-4.50) were associated with high use of PIM under both criteria. In contrast, coronary heart disease and number of comorbidities were negatively associated factors, and ischemic stroke showed criterion-dependent differences in its association with PIMs.

**Conclusion:**

The prevalence of PIMs among older Chinese outpatients with depression was remarkably high in this cohort, with associations partly different across the two criteria. The single-center retrospective design, unmeasured confounders, unavailability of renal function data, and lack of follow-up assessment warrant cautious interpretation of the results. Multidisciplinary medication review and individualized deprescribing strategies can be implemented in clinical practice for this vulnerable population.

## Introduction

With global population aging, the percentage of people aged 65 years or over, referred to as the geriatric population, has been increasing rapidly (1). From China’s Seventh National Population Census, the data revealed that the proportion of adults aged 65 and older rose from 8.9% to 13.5%, representing an increase of 4.6% over the preceding decade (2). Aging is accompanied by substantial physiological changes that increase the vulnerability of the geriatric population, making pharmacotherapeutic strategies particularly challenging (3). Due to the high prevalence of diseases in older adults, polypharmacy has emerged as a significant challenge in clinical management of these patients (4). It frequently involves the use of PIM (potentially inappropriate medication) among this population. PIM was defined as a drug in which the risk of adverse outcomes exceeds the therapeutic benefits, especially when alternative treatments with better safety or efficacy are available for the same condition (5, 6). PIM exposure in this population has been associated with elevated risks of adverse drug reactions, increased morbidity and mortality, and excessive utilization of healthcare resources (7). Therefore, it is important to monitor and reduce the prevalence of PIM use in older adults.

Depression represents one of the most prevalent mental disorders among the geriatric population, seriously affecting their daily functioning and overall well-being (8). A meta-analysis of 92 studies from low and middle-income countries showed a 38.76% prevalence of depressive disorder in adults ≥60 years (9), whereas the overall prevalence of depression among older adults in China exceeded 20% (10). Depressed patients face a significantly increased risk of developing comorbid conditions, and those with both depression and chronic disease experience greater morbidity, healthcare utilization, costs, and mortality than non-depressed patients with chronic disease (11–13). Among pharmacological strategies for managing depression in older adults, antidepressants represent the preferred first-line option (14). Nevertheless, fewer than half of older patients achieve an adequate therapeutic response to initial antidepressant therapy (15), and these agents are associated with wide-ranging side effects (16). Extensive use of antidepressants in older adults has raised concerns about misuse and an increase in the risk of PIMs, which could negatively affect treatment outcomes and impose a socioeconomic burden (17). Therefore, special attention should be paid to PIM care management in older patients with depression.

A variety of tools have been developed to screen for PIM use in older adults. The earliest expert consensus on PIMs was introduced in 1991, named the Beers criteria, which was designed by a multidisciplinary panel comprising professionals from geriatrics, pharmacy, nursing, and psychopharmacology (18, 19). These criteria have been broadly adopted in drug application surveys in communities, clinics, and hospitals in multiple countries, serving as a practical tool for managing drug-therapy in older adults (18). Updated in 2023, the criteria remain one of the most popular guidelines for the evaluation of PIM use in older people (19, 20). The Geriatrics Branch of Chinese Medical Association, in collaboration with multiple professional societies formulated a China-specific set of PIM criteria for older adults in China in 2017, the Chinese criteria were also widely used in Chinese older patients (21). Other widely used tools for identifying PIMs include the STOPP/START criteria in Europe and the PRISCUS list in Germany (22, 23).

Although PIMs have been widely studied in general older adult populations, few studies specifically address PIMs in older outpatients with depression. Some studies are primarily conducted in older inpatients (24, 25); others are limited to small samples (n < 1,000) (26, 27) or single-local criterion assessments (e.g., only the Danish List, the PRISCUS List, or the Chinese criteria) (17, 24, 28). Besides that, current studies concerning PIMs in older patients with depression are primarily conducted in older adults around the world, yet very few have been carried out in China. Of the three studies conducted in China, all used only the Chinese criteria as the sole PIM assessment tool, and only one enrolled more than 1,000 participants (28–30). Notably, for the Chinese population with depression, no authoritative Beers-based assessment has been reported, nor has any comparative study between the Beers criteria and the Chinese criteria been conducted. As we know, the Beers criteria are internationally authoritative and are the most widely used standard worldwide. However, marked discrepancies exist between the Chinese criteria and the Beers criteria. For instance, the Chinese criteria do not establish a dedicated classification for medications to be used with caution. This gap is particularly prominent for first-line antidepressants including SSRIs (selective serotonin reuptake inhibitors) and SNRIs (serotonin-norepinephrine reuptake inhibitors), which are explicitly classified as cautionary medications in the Beers criteria and carry high clinical significance for depressed patients. Therefore, the Chinese criteria may lead to under-identification of certain PIMs, particularly those involving antidepressants. Consequently, a comprehensive PIM evaluation, integrating both the Beers criteria and Chinese criteria, is urgently needed among older Chinese outpatients with depression.

Therefore, this study was the first to conduct a parallel, dual-criteria evaluation (the 2023 AGS Beers criteria and the 2017 Chinese criteria) of PIMs specifically among older Chinese outpatients with depression, and it first comprehensively quantified the effects of associated factors in this specialized population based on the Beers criteria. We described the medication use patterns in this population and investigated the prevalence of and factors associated with PIMs. These findings are expected to provide better evidence-based support specifically for the population of older Chinese depressed outpatients.

## Methods

### Study design and population

This cross-sectional study was conducted at Tongde Hospital of Zhejiang Province, a provincial tertiary psychiatric center located in Hangzhou, China. The hospital serves a diverse patient population, with an annual outpatient volume of approximately 1.53 million visits in 2024, of which approximately 210,000 involved patients aged ≥65 years. Data for this study were retrospectively extracted from the hospital’s electronic medical record system, to evaluate the prevalence and associated factors of PIMs among older outpatients with depression. We employed a consecutive census approach without applying random or stratified sampling applied. This full-coverage approach eliminates sampling error and ensures that our findings reflect the real-world spectrum of this patient population at our center.

Eligible participants comprised outpatients aged ≥65 years who presented to the facility between October 1st, 2024 and September 30th, 2025, with a documented diagnosis of depression. Depression diagnoses were established by qualified psychiatrists or geriatric physicians according to standard diagnostic criteria. Eligible patients were required to have at least one prescription medication documented during the outpatient visit and complete demographic, diagnostic, and prescription data were available in the electronic medical record system.

Patients were excluded if they met any of the following criteria: (1) incomplete or missing clinical diagnosis, defined as the absence of a documented ICD-10 code for depression or incomplete diagnostic records in the electronic medical record system; (2) incomplete prescription data, including missing information on age, sex, or comorbidities essential for PIM identification, or missing data on drug name, drug specification, dosage, administration route, administration frequency, or prescription expenditure; and (3) topical formulations only, defined as patients prescribed exclusively topical agents (such as dermatological creams, ophthalmic drops, nasal sprays, or transdermal patches without systemic absorption), as these do not contribute to systemic PIM exposure and therefore do not affect PIM assessment.

All eligible patients were consecutively enrolled in the final analysis, and no missing data imputation was required.

### Data collection

Data were systematically extracted from the hospital’s electronic medical record system and integrated prescription database by trained research personnel. Data collection captured prescription-related information including general information and medication information. General information included prescription number, department name, patient number, patient gender, patient age, clinical diagnosis and prescription expenditure. Medication information included drug name (generic name and trade name), drug specification (including the strength and dosage form of the drug), dosage, administration route, and administration frequency. When calculating the number of drugs, water for injection and topical ointment were not included.

### PIMs identification criteria

The assessment of PIM use in older patients was performed using both the 2023 AGS Beers criteria and the 2017 Chinese criteria. The 2023 AGS Beers criteria comprise five categories: potentially inappropriate medications in older adults, medications to be avoided in older adults with certain diseases or syndromes, drugs requiring caution in older adults, drug-drug interactions that should be avoided, and medications requiring dose adjustment or avoidance according to kidney function (20). Because data on renal function of older outpatients were unavailable, the criteria requiring dose adjustment or avoidance based on creatinine clearance were not applied, and PIM evaluation was limited to the other four Beers categories. The 2017 Chinese criteria defined two types of potentially inappropriate medication: potentially inappropriate medication use in older patients and potentially inappropriate medication use in older patients with a specific disease or syndrome (21). Each PIP (potentially inappropriate prescription) was assessed on the basis of the number of PIM entries, according to the aforementioned standards, any prescription containing at least one PIM was classified as one PIM-related PIP.

Two independent clinical pharmacists manually reviewed all flagged prescriptions to verify their appropriateness and exclude any false-positive detections. Any disagreements identified by the two reviewers were brought to a third professional, and were subsequently settled by collective discussion.

### Statistical analysis

Categorical variables were presented as frequencies (N) and percentages (%), χ2 test or Fisher’s exact test was used to compare categorical variables between groups. Continuous variables were expressed as mean ± standard deviation (SD) or median ± interquartile range (IQR). We assessed multicollinearity among all candidate predictors using the variance inflation factor (VIF). To identify factors associated with PIM use, univariable logistic regression analyses were first conducted. Variables with a P-value < 0.05 in the univariable analysis were then entered into a multivariable logistic regression model. Those with P < 0.05 in the multivariable model were considered significant contributors to PIM. Results of the logistic regression were reported as odds ratios (ORs) and their 95% confidence intervals (CIs). Sample size adequacy was assessed using the events-per-variable (EPV) criterion. The goodness of fit of the multivariable logistic regression models was evaluated using the Hosmer-Lemeshow test and Nagelkerke’s pseudo-R². We also performed interaction and subgroup analyses to examine whether the associations between comorbidities and PIM use were modified by other coexisting conditions. In addition, to test the robustness of our findings about the factors associated with PIMs, we conducted a sensitivity analysis by modifying the 2017 Chinese criteria incorporating SSRI- and SNRI-induced PIMs and concurrently excluding clopidogrel-induced PIMs. All tests were two-tailed, and P-value < 0.05 was considered statistically significant. Statistical analyses were performed using R software (version 4.3.1).

### Ethics approval and registration

This study was approved by the Ethics Committee of Tongde Hospital of Zhejiang Province (No. 2025-465-JY) and registered in the Chinese Clinical Trial Registry (ChiCTR2500114900). All procedures performed in this study conformed to the Helsinki Declaration and its subsequent amendments. The study’s informed consent requirement was waived owing to its retrospective nature.

## Results

### Basic clinical characteristics of patients

Based on the inclusion and exclusion criteria, a total of 3,155 older outpatients with depression were included in the final analysis. The mean age of the cohort was 74.22 ± 6.70 years, with 2,470 patients (78.29%) aged 65–79 years, and 685 patients (21.71%) aged ≥80 years. The cohort displayed a pronounced female predominance, with females constituting 67.54% (n = 2,131) of the total participants, compared to 32.46% (n = 1,024) males. Polypharmacy, defined as the concurrent prescription of 5 or more medications, was identified in 13.22% (n = 417) of the patients, with 12.65% receiving 5 to 9 medications and 0.57% receiving 10 or more. Non-polypharmacy (1 to 4 drugs) was observed in 86.78% (n = 2,738) of the cohort.

A total of 2,459 patients (77.94%) had at least one comorbidity in addition to depression. The most frequent comorbidities were ischemic stroke (10.87%), hypertension (11.00%), anxiety disorders (32.33%), and sleep disorders (35.72%). Basic characteristics in this study are presented in **Table 1**.

**Table 1:** Basic characteristics of participants.

Variables	Total (n = 3155)	2023 Beers criteria			2017 Chinese criteria		
		non-PIM group (n = 230)	PIM group (n = 2925)	P	non-PIM group (n = 1100)	PIM group (n = 2055)	P
Age, Mean ± SD	74.22 ± 6.70	75.80 ± 7.06	74.10 ± 6.66	< 0.001	74.24 ± 6.35	74.21 ± 6.89	0.932
Sex, n (%)				0.005			0.038
Male	1024 (32.46)	94 (40.87)	930 (31.79)		383 (34.82)	641 (31.19)	
Female	2131 (67.54)	136 (59.13)	1995 (68.21)		717 (65.18)	1414 (68.81)	
Drug number, Median (IQR)	2.00 (1.00, 3.00)	1.00 (1.00, 2.00)	2.00 (2.00, 4.00)	< 0.001	1.00 (1.00, 2.00)	3.00 (2.00, 4.00)	< 0.001
Cost, n (%)				0.658			0.185
<500 CNY	2379 (75.40)	168 (73.04)	2211 (75.59)		810 (73.64)	1569 (76.35)	
500–1000 CNY	589 (18.67)	48 (20.87)	541 (18.5)		216 (19.64)	373 (18.15)	
≥1000 CNY	187 (5.93)	14 (6.09)	173 (5.91)		74 (6.73)	113 (5.5)	
Number of comorbidities, n (%)				0.002			< 0.001
0	696 (22.06)	32 (13.91)	664 (22.7)		306 (27.82)	390 (18.98)	
≥1	2459 (77.94)	198 (86.09)	2261 (77.3)		794 (72.18)	1665 (81.02)	
Hypertension, n (%)	347 (11.00)	54 (23.48)	293 (10.02)	< 0.001	108 (9.82)	239 (11.63)	0.121
Atrial Fibrillation, n (%)	29 (0.92)	2 (0.87)	27 (0.92)	0.999	15 (1.36)	14 (0.68)	0.056
Heart failure, n (%)	16 (0.51)	2 (0.87)	14 (0.48)	0.327	6 (0.55)	10 (0.49)	0.825
CHD, n (%)	160 (5.07)	27 (11.74)	133 (4.55)	< 0.001	43 (3.91)	117 (5.69)	0.029
COPD, n (%)	9 (0.29)	1 (0.43)	8 (0.27)	0.494	7 (0.64)	2 (0.1)	0.011
Alzheimer, n (%)	39 (1.24)	6 (2.61)	33 (1.13)	0.06	15 (1.36)	24 (1.17)	0.635
Ischemic stroke, n (%)	343 (10.87)	49 (21.3)	294 (10.05)	< 0.001	61 (5.55)	282 (13.72)	< 0.001
Anxiety disorder, n (%)	1020 (32.33)	97 (42.17)	923 (31.56)	< 0.001	373 (33.91)	647 (31.48)	0.165
Sleep disorders, n (%)	1127 (35.72)	63 (27.39)	1064 (36.38)	0.006	245 (22.27)	882 (42.92)	< 0.001
Constipation, n (%)	143 (4.53)	16 (6.96)	127 (4.34)	0.066	35 (3.18)	108 (5.26)	0.008

Continuous variables were expressed as mean ± SD or median ± IQR. Categorical variables were presented as frequencies (N) and percentages (%). The χ2 test or Fisher’s exact test was used to compare categorical variables between groups. No missing data imputation was required. PIM, potentially inappropriate medication; IQR, interquartile range; SD, standard deviation; CNY, Chinese Yuan; CHD, coronary heart disease; COPD, chronic obstructive pulmonary disease

### Prevalence of PIMs

PIM prevalence differed substantially depending on the assessment tool. Based on the 2023 AGS Beers criteria, 92.71% of the patients were exposed to at least one PIM. As for the 2017 Chinese criteria, the prevalence was 65.13%. Differences were also observed in the number of PIMs prescribed per patient. Under the Beers criteria, 36.99% of patients received 1 PIM, 31.20% received 2 PIMs, 18.22% received 3 PIMs, and 5.39% were prescribed 4 or more PIMs. Under the Chinese criteria, the distribution was weighted toward single PIM exposure: 42.88% of patients received 1 PIM, 17.21% received 2 PIMs, 4.63% received 3 PIMs, and 0.41% received 4 or more PIMs (**Table 2**).

**Table 2:** The number of PIMs used as identified by the 2023 Beers criteria and the 2017 Chinese criteria.

Characteristics	Beers criteria		Chinese criteria	
1 PIM	1167	36.99%	1353	42.88%
2 PIMs	1013	31.20%	543	17.21%
3 PIMs	575	18.22%	146	4.63%
≥4 PIMs	170	5.39%	13	0.41%
At least 1 PIM	2,925	92.71%	2,055	65.13%

Data are presented as frequencies (n) and percentages (%), with percentages calculated based on the total study population (N = 3,155). Percentages have been rounded to two decimal places. PIM, potentially inappropriate medication.

### Most frequent PIMs

According to the 2023 AGS Beers criteria, the most frequently prescribed PIMs were quetiapine (11.41% of all PIMs), escitalopram (9.48%), sertraline (7.45%), zolpidem (6.21%), clonazepam (5.48%), venlafaxine (5.41%), mirtazapine (5.24%), estazolam (4.83%), paroxetine (4.38%), and olanzapine (4.15%). The high prevalence under this standard was largely attributable to the classification of SSRIs and SNRIs as PIMs.

**Figure 1:**
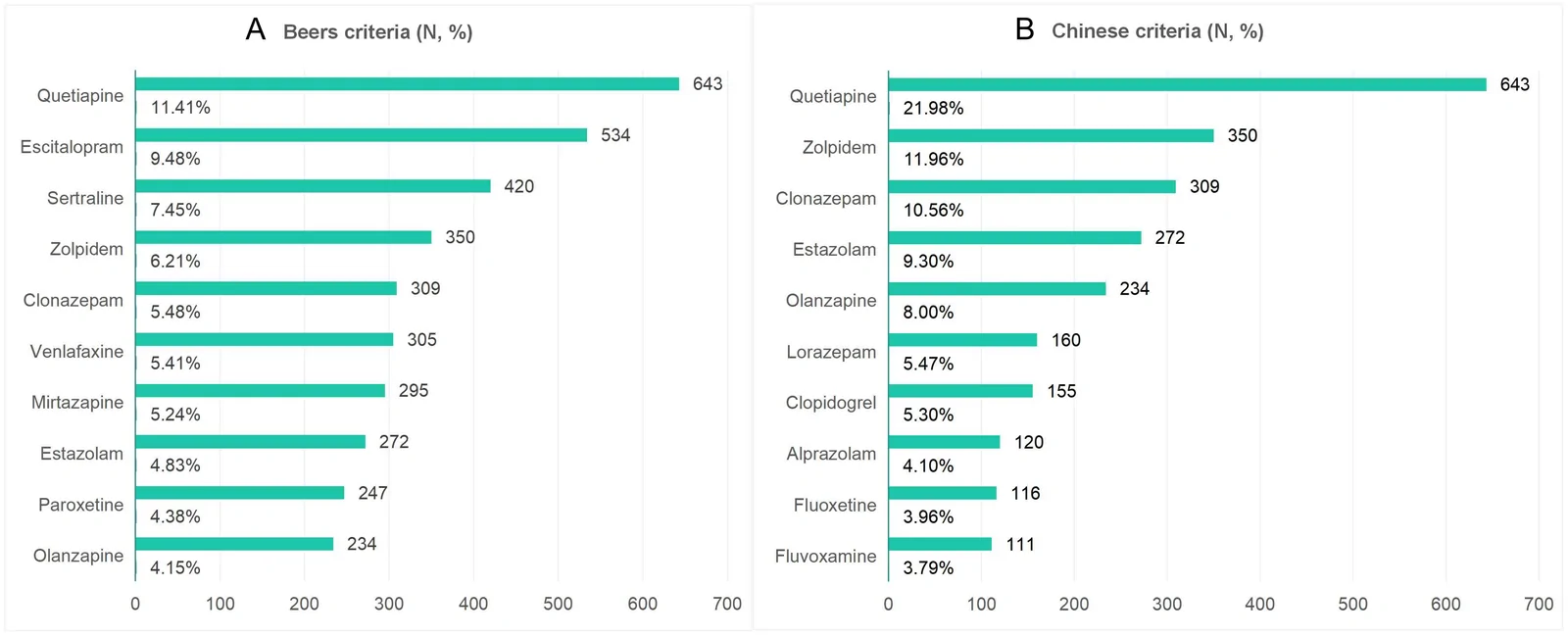
Top ten most frequently prescribed potentially inappropriate medications based on **(A)** the 2023 Beers criteria and **(B)** the 2017 Chinese criteria. Percentages represent the proportion of all potentially inappropriate medications identified by each respective criterion.

As for the 2017 Chinese criteria, the most common PIMs were: quetiapine (21.98% of all PIMs), zolpidem (11.96%), clonazepam (10.56%), estazolam (9.30%), olanzapine (8%), lorazepam (5.47%), clopidogrel (5.30%), alprazolam (4.10%), fluoxetine (3.96%), and fluvoxamine (3.79%). Atypical antipsychotics and benzodiazepines were primarily identified as PIMs in the Chinese criteria. Quetiapine ranked highest under both Criteria. The ten most frequently prescribed individual PIMs are shown in **Figure 1**.

Notably, our study also revealed significant differences between the PIMs of two criteria in the context of antidepressant medication prescribing. In our study, 14 antidepressants were identified as PIMs (5 in the top 10) under the Beers criteria, whereas only 3 were identified (2 in the top 10) under the Chinese criteria.

### Factors associated with PIMs

Multicollinearity was assessed using the VIF. As shown in **Supplementary Table 1**, all values for both models were below 2, indicating no significant multicollinearity. Multivariable logistic regression analyses were conducted to identify factors associated with PIMs according to the Beers criteria and the Chinese criteria, respectively. The results revealed distinct patterns of association depending on the criteria applied (**Table 3**).

**Table 3:** Univariable and multivariable logistic regression analysis of factors associated with PIM use.

Variable	2023 Beers criteria				2017 Chinese criteria			
	Univariable		Multivariable		Univariable		Multivariable	
	OR (95% CI)	P value	aOR (95% CI)	P value	OR (95% CI)	P value	aOR (95% CI)	P value
Age, years	0.97 (0.95~0.98)	<0.001	0.98 (0.96~1.01)	0.149	1 (0.99~1.01)	0.932	-	-
Sex, female vs. male	1.48 (1.13~1.95)	0.005	1.57 (1.16~2.12)	0.003	1.18 (1.01~1.38)	0.038	1.44 (1.2~1.73)	<0.001
Drug number	1.96 (1.69~2.28)	<0.001	2.43 (2.08~2.85)	<0.001	2.38 (2.19~2.58)	<0.001	2.51 (2.3~2.74)	<0.001
Cost, 500–1000 CNY vs. <500 CNY	0.86 (0.61~1.2)	0.363	-	-	0.89 (0.74~1.08)	0.231	-	-
Cost, ≥1000 CNY vs. <500 CNY	0.94 (0.53~1.65)	0.828	-	-	0.79 (0.58~1.07)	0.127	-	-
Number of comorbidities, ≥1 vs 0	0.55 (0.38~0.81)	0.002	0.45 (0.29~0.7)	<0.001	1.65 (1.39~1.95)	<0.001	0.53 (0.42~0.65)	<0.001
Hypertension, yes vs. no	0.36 (0.26~0.5)	<0.001	0.31 (0.21~0.46)	<0.001	1.21 (0.95~1.54)	0.122	-	-
Atrial Fibrillation, yes vs. no	1.06 (0.25~4.49)	0.935	-	-	0.5 (0.24~1.03)	0.061	-	-
Heart failure, yes vs. no	0.55 (0.12~2.43)	0.428	-	-	0.89 (0.32~2.46)	0.825	-	-
CHD, yes vs. no	0.36 (0.23~0.55)	<0.001	0.29 (0.17~0.51)	<0.001	1.48 (1.04~2.12)	0.03	0.57 (0.36~0.91)	0.019
COPD, yes vs. no	0.63 (0.08~5.04)	0.662	-	-	0.15 (0.03~0.73)	0.059	-	-
Alzheimer, yes vs. no	0.43 (0.18~1.03)	0.057	-	-	0.85 (0.45~1.64)	0.636	-	-
Ischemic stroke, yes vs. no	0.41 (0.29~0.58)	<0.001	0.27 (0.18~0.42)	<0.001	2.71 (2.03~3.61)	<0.001	1.7 (1.2~2.41)	0.003
Anxiety disorder, yes vs. no	0.63 (0.48~0.83)	0.001	0.78 (0.57~1.07)	0.124	0.9 (0.77~1.05)	0.165	-	-
Sleep disorders, yes vs. no	1.52 (1.12~2.04)	0.006	1.79 (1.27~2.51)	0.001	2.62 (2.22~3.1)	<0.001	3.67 (2.99~4.5)	<0.001
Constipation, yes vs. no	0.61 (0.35~1.04)	0.069	-	-	1.69 (1.14~2.49)	0.008	0.74 (0.44~1.24)	0.247

Data are presented as OR with 95% CI, aOR represents adjusted odds ratios from multivariable models. Variables with P < 0.05 in univariable analysis were entered into the multivariable logistic regression model. Multivariable models were adjusted for age, sex, number of medications, and comorbidities that showed statistical significance in univariable analysis. PIM, potentially inappropriate medication; CNY, Chinese Yuan; CHD, coronary heart disease; COPD, chronic obstructive pulmonary disease; OR, odds ratio; CI, confidence interval; aOR, adjusted odds ratio.

After adjusting for potential confounders, the analysis revealed several factors associated with PIM exposure based on the 2023 AGS Beers criteria. Female sex was identified as an independent factor positively associated with PIM use (aOR [adjusted OR] = 1.57, 95% CI: 1.16-2.12, P = 0.003). Polypharmacy (aOR = 2.43, 95% CI: 2.08-2.85, P < 0.001) and sleep disorders (aOR = 1.79, 95% CI: 1.27-2.51, P = 0.001) showed similarly positive associations. Conversely, number of comorbidities (aOR = 0.45, 95% CI: 0.29-0.70, P < 0.001), hypertension (aOR = 0.31, 95% CI: 0.21-0.46, P < 0.001), CHD (coronary heart disease) (aOR = 0.29, 95% CI: 0.17-0.51, P < 0.001), and ischemic stroke (aOR = 0.27, 95% CI: 0.18-0.42, P < 0.001) demonstrated negative associations with PIMs.

Consistent with the Beers model, the 2017 Chinese criteria also demonstrated that female sex (aOR = 1.44, 95% CI: 1.20-1.73, P < 0.001), number of prescribed medications (aOR = 2.51, 95% CI: 2.30-2.74, P < 0.001), and sleep disorders (aOR = 3.67, 95% CI: 2.99-4.50, P < 0.001) were independently positively associated with PIM use, with sleep disorders emerging as the most prominent factor. Notably, ischemic stroke (aOR = 1.70, 95% CI: 1.20-2.41, P = 0.003) was also positively associated under the Chinese criteria, which exhibited opposite associations compared with those under the Beers criteria. Conversely, number of comorbidities (aOR = 0.53, 95% CI: 0.42-0.65, P < 0.001) and CHD (aOR = 0.57, 95% CI: 0.36-0.91, P = 0.019) were negatively associated with PIM use.

Sample size adequacy was assessed using the EPV criterion: the Beers criteria model included 9 predictor variables (EPV = 25.6; 230 events in the smaller group ÷ 9), and the Chinese criteria model included 7 predictor variables (EPV = 157.1; 1,100 ÷ 7) both exceeding the recommended threshold of EPV > 10 (31), indicating that the sample size was sufficient for the multivariable logistic regression analyses. Model diagnostics indicated acceptable fit for both multivariable models. The Hosmer-Lemeshow test showed no significant lack of fit (Beers criteria model: χ² = 16.49, P = 0.063; Chinese criteria model: χ² = 11.26, P = 0.166). We calculated Nagelkerke’s pseudo-R² as a measure of model fit, obtaining a value of 0.225 for the Beers criteria model and 0.243 for the Chinese criteria model, both of which indicate an acceptable moderate model fit.

Because hypertension, CHD, and ischemic stroke are deeply interrelated cardiovascular conditions, pairwise interaction terms among these three conditions were examined in the multivariable models and tested their significance using the likelihood ratio test. In the Beers criteria model, adding interaction terms significantly improved model fit (χ² = 14.67, P = 0.002). The hypertension × CHD (P = 0.003) and hypertension × ischemic stroke (P = 0.008) interactions were both significant, whereas CHD × ischemic stroke was not (P = 0.582). Given these significant interactions, subgroup analyses showed that hypertension was strongly associated with lower odds of PIM use in patients without CHD (OR = 0.21, 95% CI: 0.14-0.33) or without ischemic stroke (OR = 0.19, 95% CI: 0.12-0.29), but this association was attenuated and non-significant in patients with CHD or ischemic stroke (OR = 0.76, 95% CI: 0.31-1.88 for both), as shown in **Supplementary Table 2**. The two significant interaction terms were retained in the final model, and main-effect estimates were essentially unchanged after their inclusion (**Supplementary Table 3**). In the Chinese criteria model, we tested the interaction between the two cardiovascular conditions retained in the model (CHD × ischemic stroke), which was not statistically significant (P = 0.564), and the results of likelihood ratio test were χ² = 0.339, P = 0.560. These results suggest that there is no significant interaction between CHD and ischemic stroke under the Chinese criteria.

We also conducted a sensitivity analysis, with the results presented in **Supplementary Tables 4, S5**. Our analysis indicated that incorporating SSRI- and SNRI-induced PIMs and concurrently excluding clopidogrel-induced PIMs in the Chinese criteria, resulted in consistent OR effect directions for ischemic stroke across both the Beers criteria and the Chinese criteria.

## Discussion

This cross-sectional study, based on data from a provincial tertiary psychiatric hospital, systematically evaluated the prevalence of PIMs in older Chinese outpatients with depression using both the 2023 Beers criteria and the 2017 Chinese criteria. Our cohort demonstrated a PIM prevalence of 92.71% under the 2023 Beers criteria and 65.13% under the Chinese criteria. In comparison with other Beers-based studies, a Spanish study of older adults with depressive disorder reported a PIM prevalence of 72.76% under the Beers criteria and 89% under the STOPP criteria, slightly lower than our Beers-based estimate (32). This difference may be attributable to cross-country variations in patient demographics and prescribing practices. A multicenter study across seven Chinese cities using the 2017 Chinese criteria reported a PIM prevalence of 50.42% among 40,516 older outpatient prescriptions for depression, which was lower than our Chinese-criteria estimate of 65.13% (28). Compared with the broader primary and secondary care settings in the multicenter sample, our center, a provincial-level psychiatric hospital, exhibited higher psychiatric complexity and more intensive psychotropic polypharmacy. This high prevalence of PIMs among older outpatients with depression indicates the need for greater attention to the management of geriatric depression.

The prevalence of Beers criteria-based PIM use in this cohort was 92.71%, which was higher (by 27.58%) than Chinese criteria-based. The high PIM prevalence in Beers criteria and the 27.58% gap in PIM prevalence between the two criteria are probably due to the variations in the medical guideline development, regional clinical focus, and the evolving perception of psychopharmacological risk. The 2023 AGS Beers criteria designate all SSRIs and SNRIs as medications requiring caution in older adults due to their association with hyponatremia, falls, fractures, and risk of bleeding (20, 33), whereas the 2017 Chinese criteria do not include these agents as PIMs (21). Antidepressants, particularly SSRIs such as escitalopram and sertraline, and SNRIs such as venlafaxine, were still the main treatment for patients with moderate to severe depression, the recommended agents are simultaneously designated as potentially inappropriate in the Beers criteria (15). Under the Beers criteria, 14 antidepressants were listed as PIMs, including escitalopram, sertraline, venlafaxine and other agents. In contrast, the Chinese criteria’s exclusion of most SSRIs reflected the divergence between AGS developed criteria and the Chinese prescribing context. In addition, a study noted that 30 medications listed in the Beers criteria were simply unavailable in China, further limiting direct comparability (34). Nevertheless, the high prevalence of Beers criteria-based PIMs should not be interpreted as indicating that these medications are universally inappropriate for older adults. It reflects the need for enhanced clinical monitoring and risk assessment, rather than discontinuation or replacement of guideline-recommended antidepressant therapy. In patients with moderate-to-severe depression, the potential therapeutic benefits of antidepressant treatment may outweigh the associated risks, and treatment decisions should therefore be individualized according to the patient’s clinical condition, comorbidities, and overall risk-benefit profile.

Quetiapine ranked first under both criteria, accounting for 11.41% of all Beers-flagged PIMs and 21.97% of Chinese-criteria PIMs. This predominance may be attributable to two distinct clinical indications, quetiapine is widely used as adjunctive or augmentation therapy for treatment-resistant depression in older adults, and low-dose quetiapine has been increasingly adopted off-label as a sedative-hypnotic agent, particularly in patients with comorbid sleep disorders (35). Both criteria flagged antipsychotics due to increased risks of stroke, cognitive decline, and mortality in older patients, risks established in randomized controlled trials largely involving dementia populations (20, 21). Consequently, clinicians managing depressed older patients on quetiapine should explicitly weigh these risks, minimize the dose, and schedule regular reassessments.

Benzodiazepines (clonazepam, lorazepam, estazolam, alprazolam) and the Z-drug zolpidem collectively dominated the PIM lists under both criteria after antipsychotics, consistent with the high co-prevalence of sleep disorders (35.72%) in this cohort. Sleep disorders affected the majority of older adults with depression and represent a primary indication for benzodiazepine prescribing (36, 37), despite both the Beers criteria and the Chinese criteria caution strongly against benzodiazepine use in this population due to risks of falls, fractures, cognitive impairment, and the development or acceleration of dementia (20, 21). Previous studies have similarly identified benzodiazepines as among the most frequently prescribed medications, and our findings in a depression cohort align with this pattern (28).

Female sex, polypharmacy, and sleep disorders were positively associated with PIM use among older Chinese outpatients with depression under both the Beers and the Chinese criteria in this study. Female sex was a factor associated with PIM use (Beers criteria: OR = 1.57; Chinese criteria: OR = 1.44), which is consistent with a previous study conducted in Spain (31). However, two other studies found no significant sex difference in PIM use among older patients with depression (17, 38). Thus, our results provide complementary evidence on this debated topic. This can be partly explained by the fact that older women have higher rates of anxiety, insomnia, depression, and urinary incontinence compared with men-conditions that are common indications for drugs frequently classified as PIMs, including benzodiazepines, anticholinergics, and antipsychotics (39). Polypharmacy was among the strongest predictors of PIM use under both criteria in our study, with ORs of 2.43 (Beers criteria) and 2.51 (Chinese criteria), consistent with findings reported in both French and Chinese studies conducted in older populations with depression (28, 40). As the number of prescribed medications in a patient’s regimen increases, the statistical probability that at least one of those agents will be classified as a PIM rises accordingly (41). Sleep disorders, ranking among the most prevalent comorbidities in the cohort, emerged as a factor associated with PIM use in older depressive population (Beers criteria: OR = 1.79; Chinese criteria: OR = 3.67). Our finding is consistent with a Chinese study that also identified comorbid sleep disorders as a predictor of PIM prescribing in older outpatients with depression (OR = 2.808) (28). The pharmacological agents most commonly used to manage sleep disorders in this population (including benzodiazepines, Z-hypnotics, and low-dose quetiapine) are classified as PIMs under both criteria, thereby potentially linking sleep disorders to elevated PIM exposure (20, 21). These findings suggest that medication review should be prioritized for older patients receiving multiple medications and those with coexisting sleep disorders. For sedative and hypnotic medications are often prescribed for sleep issues and may increase the overall medication burden and cause adverse effects in this population.

We also observed that the number of comorbidities was negatively associated with PIM use under both criteria (Beers: aOR = 0.45; Chinese: aOR = 0.53). Notably, this negative association has been documented in several other independent studies (42–44). We hypothesize that this observation may be explained by the adjustment for confounding factors: after controlling for individual comorbidities and medication count, the residual variance captured by the composite “≥1 comorbidity” variable may be predominantly driven by patients with multiple mild conditions who receive fewer PIM-indicated drugs, thereby yielding the observed negative association. Patients with comorbidities receive more intensive care and frequent medication reviews, which may promote rational prescribing and reduce inappropriate use. In contrast, patients with depression alone often lack such scrutiny. Furthermore, in multimorbid patients, depression may be viewed as secondary, leading clinicians to prefer non-pharmacological interventions or safer antidepressants. Nevertheless, the exact mechanisms underlying this finding warrant further investigation in future studies.

Our findings also identified two interesting factors that were significantly associated with PIM use. One factor was ischemic stroke, negatively associated with Beers-based PIM use but positively associated with Chinese-criteria PIM use. Under the Chinese criteria, clopidogrel, a PIM for older patients owing to hematologic risks, was widely used for secondary stroke prevention, thereby increasing PIM prevalence in patients with ischemic stroke (21, 45). The Beers criteria, by contrast, did not designate clopidogrel as a PIM, precluding this source of flagging (20). Additionally, because the Beers criteria classified standard first-line antidepressants (SSRIs and SNRIs) as PIMs, prescriber caution may reduce antidepressant use in stroke patients, consequently lowering their Beers-based PIM burden. And this result was further supported by sensitivity analysis by incorporating SSRI- and SNRI-induced PIMs and concurrently excluding clopidogrel-induced PIMs under Chinese criteria. These findings illustrated that the two criteria were developed with distinct regional pharmacopeias and prescribing conventions, and a combined evaluation using multiple criteria may therefore be needed.

The other factor was CHD, which was negatively associated with PIM use among older outpatients with depression under both criteria, which was also supported by another similar study (28). CHD acted as a negative association against PIMs, possibly because it prompted physicians and patients to adopt more conservative and evidence-based medication strategies, thereby reducing unnecessary polypharmacy and inadvertently lowering the prescription rate of PIMs. Moreover, similar negative associations can also be seen in ischemic stroke and hypertension under single criteria, which might be related to the fact that hypertension, CHD, and ischemic stroke are interrelated cardiovascular conditions with potential interaction effects. This was supported by the results of our interaction analysis, with the OR >1 of hypertension × CHD (OR = 5.71, 95% CI: 1.84-17.73, P = 0.003) and hypertension× ischemic stroke (OR = 3.78, 95% CI: 1.39-10.28, P = 0.009) under the Beers criteria (**Supplementary Table 3**).

The primary strength of this investigation is that it provides the first Beers-based PIM assessment (including prevalence, most frequent PIMs, and associated factors) among Chinese older outpatients with depression, and also performs a comparative analysis between the Beers criteria and the Chinese criteria. Rather than relying on a single evaluative framework, this comparative design enabled a multi-dimensional assessment in real-world clinical practice. Furthermore, comparisons between the two criteria revealed several interesting factors associated with PIMs in this specific population of older Chinese outpatients with depression. Female sex, polypharmacy, and sleep disorders emerged as positive associated factors; CHD as a negative associated factor; and ischemic stroke showed opposite directions of association under the two criteria. This finding suggests that reliance on a single criterion may be insufficient for accurately identifying PIMs in older Chinese outpatients with depression, and can provide a valuable reference to help physicians improve medication management. Additionally, some factors, such as CHD, ischemic stroke, and hypertension were negatively associated with PIM use under the Beers criteria among older outpatients with depression. Our interaction and subgroup analyses revealed significant interactions between hypertension and both CHD and ischemic stroke, which may partly explain their negative associations with PIM use. Finally, this study was conducted at a provincial psychiatric center, enrolling a large, well-characterized sample of older depressive outpatients from diverse geographic and clinical settings across the province. This institutional context yields a patient population with broader diagnostic, comorbidity, and pharmacological profiles than typically seen in community or primary care settings. Consequently, the Beers and Chinese criteria are complementary and should be interpreted alongside clinical context and individualized goals; medication review should be prioritized for older depressed patients who are female, on multiple medications, or have comorbid sleep disorders, with particular attention to quetiapine, benzodiazepines, and sedative-hypnotics.

This study had several limitations. First, although the study was conducted at a tertiary general hospital with a provincial psychiatric center and included patients from diverse provincial settings, findings from this single tertiary psychiatric center should be generalized cautiously to primary care or community outpatient settings. Patients in this setting may present with greater psychiatric complexity and more intensive psychotropic polypharmacy than those managed in primary care. Second, as this was a retrospective study using outpatient electronic medical records, depression severity, frailty status, and cognitive function could not be captured in our dataset, despite their likely influence on both prescribing patterns and PIM use; patients with more severe depression or cognitive impairment may be more likely to receive multiple psychotropic agents, potentially biasing our estimates. Third, renal function data were also unavailable in outpatient records, precluding assessment of PIMs requiring dose adjustment based on creatinine clearance, which may have led to an underestimation of PIM prevalence under the Beers criteria. Fourth, the absence of a follow-up assessment further precluded evaluation of the association between PIM use and clinical outcomes such as efficacy, adverse drug events, falls, and hospitalization. Multicenter studies incorporating standardized assessments of depression severity, frailty, and cognitive function across a broader range of healthcare settings are required to validate and extend our findings.

## Conclusion

This investigation provided the first comparative PIM assessment between the Beers criteria and the Chinese criteria among Chinese older outpatients with depression, and demonstrated a high prevalence of PIMs in this population. Polypharmacy, female sex, and comorbid sleep disorders were consistently associated with PIM use across both criteria. Targeted medication review, multidisciplinary pharmacist involvement, and prescriber education may help optimize prescribing and improve medication safety in this vulnerable population.

## Data Availability

The raw data supporting the conclusions of this article will be made available by the authors, without undue reservation.
