# Supplementary material for: Potentially inappropriate medications in older outpatients with depression: a cross-sectional study comparing the Beers criteria and the Chinese criteria
Source: Front Psychiatry. 2026 Sep 3;17:1915277. doi: 10.3389/fpsyt.2026.1915277 (PMC13582601; doi:10.3389/fpsyt.2026.1915277)
Supplement: Supplementary file 1 [file SupplementaryFile1.pdf]

Supplementary Table S1. Multicollinearity diagnostic results of 2023 Beers criteria and 2017 Chinese criteria

Predictor	GVIF	GVIF^(1/(2*Df))
**2023 Beers criteria**		
Age	1.115877	1.056351
Sex	1.152328	1.073465
Drug number	1.046159	1.022819
Cost	1.028962	1.007163
Number of Comorbidity	1.302953	1.141470
Hypertension	1.189600	1.090688
Atrial Fibrillation	1.053202	1.026256
Heart failure	1.114977	1.055925
CHD	1.281822	1.132176
COPD	1.019339	1.009623
Alzheimer	1.151433	1.073048
Ischemic stroke	1.154117	1.074298
Anxiety disorder	1.127851	1.062003
Sleep disorders	1.136451	1.066044
Constipation	1.195930	1.093586
**2017 Chinese criteria**		
Age	1.079122	1.038808
Sex	1.189505	1.090644
Drug number	1.035938	1.01781
Cost	1.026363	1.006527
Number of Comorbidity	1.466799	1.211115
Hypertension	1.172114	1.082642
Atrial Fibrillation	1.098507	1.048097
Heart failure	1.11802	1.057365
CHD	1.181804	1.087108
COPD	1.066043	1.032493

Alzheimer	1.083248	1.040792
Ischemic stroke	1.079921	1.039192
Anxiety disorder	1.121891	1.059194
Sleep disorders	1.27278	1.128175
Constipation	1.121536	1.059026

CHD, Coronary heart disease; COPD, chronic obstructive pulmonary disease; GVIF, Generalized Variance Inflation Factor; GVIF^(1/(2*Df)) is the degrees-of-freedom-adjusted value, with values greater than 2 indicating problematic collinearity. All values are below 2, suggesting no severe multicollinearity among factors.

Supplementary Table S2. Subgroup analysis of the association between hypertension and potentially inappropriate medication use according to ischemic stroke and coronary heart disease status.

Subgroup	Total, n	PIM, n (%)	aOR (95% CI)	P value	P for interaction
Without IS					0.002
Hypertension, no	2542	2404 (94.6)	Ref		
Hypertension, yes	270	227 (84.1)	0.19 (0.12-0.29)	<0.001	
With IS					
Hypertension, no	266	228 (85.7)	Ref		
Hypertension, yes	77	66 (85.7)	0.76 (0.31-1.88)	0.558	
Without CHD					<0.001
Hypertension, no	2725	2567 (94.2)	Ref		
Hypertension, yes	270	225 (83.3)	0.21 (0.14-0.33)	<0.001	
With CHD					
Hypertension, no	83	65 (78.3)	Ref		
Hypertension, yes	77	68 (88.3)	1.16 (0.38-3.53)	0.794	

OR, odds ratio; CI, confidence interval; CHD, Coronary heart disease; IS, Ischemic stroke; PIM, potentially inappropriate medication.

Supplementary Table S3. Multivariable logistic regression analysis incorporating interaction effects under the Beers criteria

Variable	aOR (95% CI)	P value
Age, years	0.99 (0.96-1.01)	0.191
Sex, female vs. male	1.52 (1.12-2.06)	0.007
Drug number	2.45 (2.10-2.87)	<0.001
Number of Comorbidity, ≥1 vs 0	0.49 (0.31-0.77)	0.002
Hypertension, yes vs. no	0.18 (0.11-0.28)	<0.001
CHD, yes vs. no	0.17 (0.08-0.33)	<0.001
Ischemic stroke, yes vs. no	0.22 (0.14-0.36)	<0.001
Anxiety disorder, yes vs. no	0.81 (0.59-1.11)	0.185
Sleep disorders, yes vs. no	1.73 (1.23-2.43)	0.002
Hypertension × CHD (interaction)	5.71 (1.84-17.73)	0.003
Hypertension × IS (interaction)	3.78 (1.39-10.28)	0.009

OR, odds ratio; CI, confidence interval; CHD, Coronary heart disease; IS, Ischemic stroke

Supplementary Table S4. Demographic and clinical characteristics of patients under the 2017 Chinese criteria by incorporating SSRI/SNR-induced PIMs and excluding clopidogrel-induced PIMs

Characteristics	Total (n = 3155)	2017 Chinese criteria		
		PIM group (n = 304)	Non-PIM group (n = 2851)	P value
Age, Mean ± SD	74.22 ± 6.70	75.35 ± 7.04	74.10 ± 6.66	0.002
Sex, n (%)				0.065
Male	1024 (32.46)	113 (37.17)	911 (31.95)	
Female	2131 (67.54)	191 (62.83)	1940 (68.05)	
Drug number, Median (IQR)	2.00 (1.00, 3.00)	1.00 (1.00, 3.00)	2.00 (2.00, 3.00)	< 0.001
Cost, n (%)				0.88
<500 CNY	2379 (75.40)	226 (74.34)	2153 (75.52)	
500-1000 CNY	589 (18.67)	60 (19.74)	529 (18.55)	
≥1000 CNY	187 (5.93)	18 (5.92)	169 (5.93)	
Number of Comorbidity, n (%)				< 0.001
0	2459 (77.94)	270 (88.82)	2189 (76.78)	
≥1	696 (22.06)	34 (11.18)	662 (23.22)	
Hypertension, n (%)	347 (11.00)	82 (26.97)	265 (9.29)	< 0.001
Atrial Fibrillation, n (%)	29 (0.92)	7 (2.3)	22 (0.77)	0.017
Heart failure, n (%)	16 (0.51)	3 (0.99)	13 (0.46)	0.195
CHD, n (%)	160 (5.07)	48 (15.79)	112 (3.93)	< 0.001
COPD, n (%)	9 (0.29)	1 (0.33)	8 (0.28)	0.599
Alzheimer, n (%)	39 (1.24)	5 (1.64)	34 (1.19)	0.419
Ischemic stroke, n (%)	343 (10.87)	57 (18.75)	286 (10.03)	< 0.001
Anxiety disorder, n (%)	1020 (32.33)	113 (37.17)	907 (31.81)	0.058
Sleep disorders, n (%)	1127 (35.72)	73 (24.01)	1054 (36.97)	< 0.001
Constipation, n (%)	143 (4.53)	19(6.25)	124 (4.35)	0.13

PIM, potentially inappropriate medication; IQR: interquartile range; SD: standard deviation; CNY, Chinese Yuan; CHD, coronary heart disease; COPD, chronic obstructive pulmonary disease；

Supplementary Table S5. Univariable and multivariable logistic regression analysis of factors for PIM under the Chinese criteria by incorporating SSRI/SNR-induced PIMs and excluding clopidogrel-induced PIMs

Variable	2017 Chinese criteria			
	Univariable		Multivariable	
	OR (95% CI)	P value	aOR (95% CI)	P value
Age, years	0.97 (0.96~0.99)	0.002	1.01 (0.99-1.03)	0.599
Sex, female vs. male	1.26 (0.99~1.61)	0.065		
Drug number	1.28 (1.17~1.4)	<0.001	1.64 (1.49-1.81)	<0.001
Cost, 500-1000 CNY vs. <500 CNY	0.93 (0.69~1.25)	0.613		
Cost, ≥1000 CNY vs. <500 CNY	0.99 (0.59~1.63)	0.955		
Number of Comorbidity, ≥1 vs 0	0.42 (0.29~0.6)	<0.001	0.34 (0.23-0.5)	<0.001
Hypertension, yes vs. no	0.28 (0.21~0.37)	<0.001	0.28 (0.2-0.4)	<0.001
Atrial Fibrillation, yes vs. no	0.33 (0.14~0.78)	0.011	1.04 (0.37-2.97)	0.937
Heart failure, yes vs. no	0.46 (0.13~1.62)	0.227		
CHD, yes vs. no	0.22 (0.15~0.31)	<0.001	0.17 (0.11-0.27)	<0.001
COPD, yes vs. no	0.85 (0.11~6.84)	0.881		
Alzheimer, yes vs. no	0.72 (0.28~1.86)	0.499		
Ischemic stroke, yes vs. no	0.48 (0.35~0.66)	<0.001	0.4 (0.28-0.57)	<0.001

PIM, potentially inappropriate medication; CNY, Chinese Yuan; CHD, Coronary heart disease; COPD, chronic obstructive pulmonary disease; OR, odds ratio; CI, confidence interval; aOR, adjusted odds ratio
